# The Appropriateness of the Helical Axis Technique and Six Available Cardan Sequences for the Representation of 3-D Lead Leg Kinematics During the Fencing Lunge

**DOI:** 10.2478/hukin-2013-0020

**Published:** 2013-07-05

**Authors:** Jonathan Sinclair, Paul J Taylor, Lindsay Bottoms

**Affiliations:** 1 Division of Sport Exercise and Nutritional Sciences, University of Central Lancashire.; 2 School of Psychology University of Central Lancashire.; 3 School of Health, Sport and Bioscience, University of East London.

**Keywords:** Cardan sequence, Euler angle, helical, fencing lunge

## Abstract

Cardan/Euler angles represent the most common technique for the quantification of segmental rotations. Cardan angles are influenced by their ordered sequence, and sensitive to planar-cross talk from the dominant rotation plane, which may affect the angular parameters. The International Society of Biomechanics (ISB) currently recommends a sagittal, coronal, and then transverse (XYZ) ordered sequence, although it has been proposed that when quantifying non-sagittal rotations this may not be the most appropriate technique. This study examined the influence of the helical and six available Cardan sequences on lower extremity three-dimensional (3-D) kinematics of the lead leg during the fencing lunge. Kinematic data were obtained using a 3-D motion capture system as participants completed simulated lunges. Repeated measures ANOVAs were used to compare discrete kinematic parameters, and intraclass correlations were also utilized to determine evidence of planar crosstalk. The results indicate that in all three planes of rotation, peak angle and range of motion angles using the YXZ and ZXY sequences were significantly greater than the other sequences. It was also noted that the utilization of the YXZ and ZXY sequences was associated with the strongest correlations from the sagittal plane, and the XYZ sequence was found habitually to be associated with the lowest correlations. It appears that for accurate representation of 3-D kinematics of the lead leg during the fencing lunge, the XYZ sequence is the most appropriate and as such its continued utilization is encouraged.

## Introduction

The most common technique within biomechanics for the quantification of 3-D kinematics is the calibrated anatomical systems technique (CAST), whereby a rigid segment axis is computed with respect to another via independent angles known as Cardan or Euler angles ( Schache et al., 2001 ). Segmental rotations which produce the resultant joint angles are considered to occur about the orientation of the segment co-ordinate system. The representation of Euler angles is obtained via an ordered sequence of rotations ( Schache et al., 2001 ; Sinclair et al., 2012 ).

It has been observed previously that altering the order of this sequence of rotations can significantly influence the 3-D angular kinematic patterns ( Thewlis et al., 2008 ; Sinclair et al., 2012 ). The International Society of Biomechanics (ISB) currently recommends lower extremity kinematics being quantified by means of an XYZ Cardan sequence of rotations, whereby X represents sagittal plane rotation, Y represents coronal plane rotation and Z represents transverse plane rotations ( Wu and Cavanagh, 1995 ). This recommendation was developed around the assumption that it is most empirically meaningful for the first rotation to represent the axis with the greatest range of motion. However, given the dominance of sagittal plane angulation during most sporting movements, it has been observed that the first rotation can impinge on the angular waveforms of the coronal and transverse planes in a phenomenon known as planar cross-talk. As such it has been proposed in more recent times that for certain movements the XYZ sequence of rotations may not be the most appropriate technique for the calculation of non-sagittal angular kinematics.

Helical axis angles can also be used to quantify segmental rotations ( Woltring et al., 1985 ). Helical angles involve the definition of both a position and orientation vector and movement from a reference position is described in terms of rotation along a single projected axis ( Sinclair et al., 2012 ). This method has been advocated by some because of lack of sequence dependency and it is not being susceptible to gimbal lock, however it is seldom utilized as it does not provide an empirically meaningful anatomical representation ( Hamill and Selbie, 2004 ).

The lunge is a fundamental offensive fencing technique and is used extensively within all three fencing disciplines: foil, épée and sabre (Sinclair et al., 2010). To score, a fencer lunges from an en guard position, quickly closing the distance to the opponent, and strikes the opponent with their weapon. Unlike the forward lunge common in many other sports, the fencing lunge maintains the perpendicular orientation of the feet, the sole of the non-leading foot remains planted on the ground, and the leading leg extends forcefully and almost completely (Paul et al., 2012). In fencing, a powerful lunge is key to a successful touch (score). Therefore, given the complexity of the movement, and its importance as an attacking movement for fencers, the correct interpretation of the movement is essential for future kinematics analyses.

A select number of investigations have examined the influence of segmental kinematic calculations on the representation of angular profiles ( Schache et al., 2001 ; Sinclair et al., 2012 ; Thewlis et al., 2008 ; Lees et al., 2010) yet the most appropriate method for the representation of lower extremity lead leg 3-D kinematics during the fencing lunge remains unknown. This study therefore investigates the influence of the 6 available Cardan sequences on lower extremity joint kinematic parameters, on planar cross-talk, in the sagittal, coronal and transverse planes during the fencing lunge.

## Material and Methods

### Participants

Fourteen participants (nine males and five females) volunteered to take part in this study (age = 26.21 ± 1.25 years; height = 175.7 ± 6.2 cm; mass = 75.6 ± 8.2 kg). All were free from musculoskeletal pathology at the commencement of data collection and provided written informed consent in accordance with the declaration of Helsinki. An a priori statistical power analysis was conducted in order to reduce the likelihood of a type II error and determine the minimum number of participants needed for this investigation. It was found that the sample size was sufficient to provide more than 80% statistical power. Ethical approval for this project was obtained from the School of Psychology ethics committee.

### Procedure

An eight camera motion analysis system (QualisysTM Medical AB, Göteborg, Sweden) captured kinematic data at 200 Hz from each participant. Calibration of the motion analysis system was performed before each data collection session. Only calibrations which produced average residuals of less than 0.85 mm for each camera for a 750.5 mm wand length and points above 4000 were accepted prior to data collection.

The marker set used for the study was based on the CAST technique ( Cappozzo et al., 1995 ). In order to define the anatomical and technical reference frames of the pelvis, right thigh, shank and foot a static reference trial was captured with each participant in the anatomical position. This allowed the positions of the anatomical markers to be referenced in relation to tracking clusters (see below). Retro-reflective markers were attached to the pelvis, right-thigh, right-shank and right-foot in the following locations: 1st and 5th metatarsal heads, medial and lateral malleoli, calcaneus, medial and lateral epicondyle of the femur, greater trochanter of the right leg, iliac crest, anterior superior iliac spines (ASIS) and posterior superior iliac spines (PSIS). The hip joint centre was defined using the Bell et al. (1989) equations via on the positions of the ASIS and PSIS markers. Tracking clusters were positioned on the right thigh and shank. Clusters were comprised of four 19mm spherical reflective markers mounted to a thin sheath of lightweight carbon fiber with a length to width ratio of 1.5-1, in accordance with the Cappozzo et al. (1997) recommendations.

### Data processing

Dynamic movement trials were processed using Qualisys Track Manager in order to label anatomical and tracking markers, following which they were exported as C3D files. 3-D Kinematic parameters were quantified using Visual 3-D (C-Motion Inc, Germantown, USA) and filtered at 12 Hz using a zero-lag low pass Butterworth 4th order filter. This was determined as being the frequency at which 95% of the signal power was contained below. Angles were created using the helical method and XYZ, XZY, YXZ, YZX, ZXY and ZYX rotation Cardan sequences referenced to co-ordinate systems about the proximal end of the segment, where X =sagittal; Y = coronal and Z = transverse plane rotations.

### Statistical Analyses

Descriptive statistics including means and standard deviations were calculated for each condition. Differences in sagittal coronal and transverse plane peak angles and ranges of motion were examined using repeated measures ANOVA’s with significance accepted at the p≤0.05 level. Appropriate post-hoc analyses were conducted using a Bonferroni correction to control for type I error. Effect sizes were calculated using an η2. As the lunge movement has an important random component intra-class correlations were utilized to compare between sagittal, coronal and transverse plane waveforms using the seven different methods. Furthermore, sagittal plane angles from all three joints were also correlated with the associated coronal and transverse plane waveforms in order to identify evidence of planar cross-talk. All statistical procedures were conducted using SPSS 19.0 (SPSS Inc, Chicago, USA)..

## Results

### Hip

**Figure 1:**
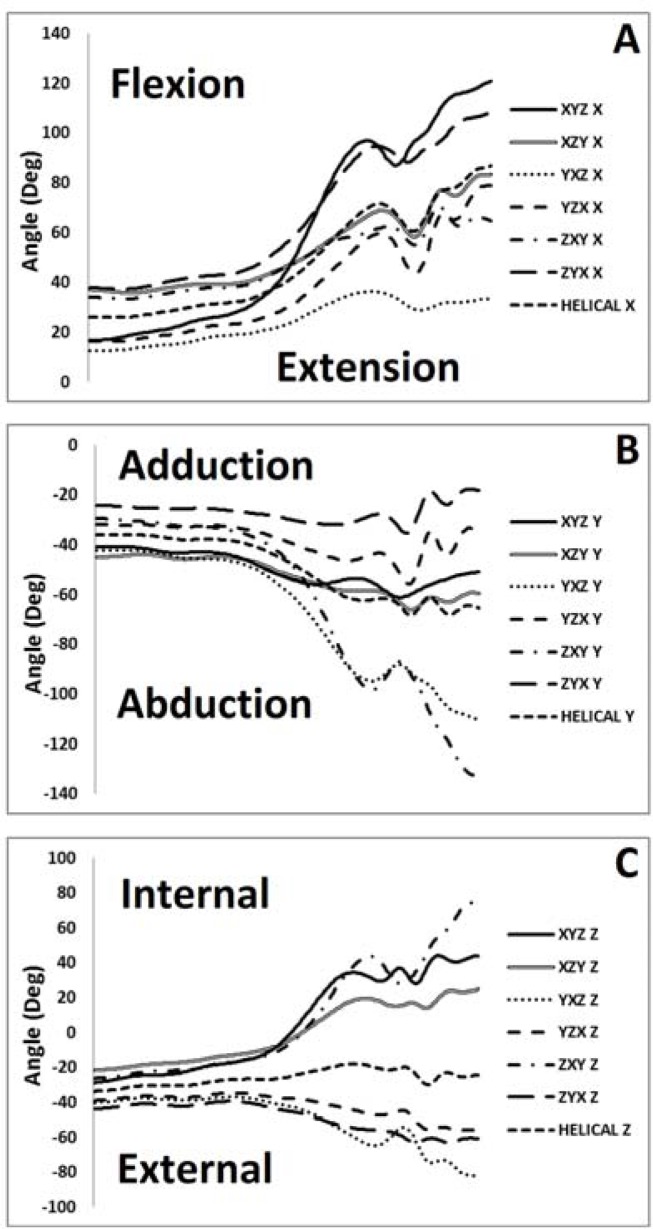
*
Hip joint kinematics in the a. sagittal, b. coronal, and c. transverse plane as a function of Cardan sequence
*

In the sagittal plane significant peak angle F (2.54, 35.07) =64.72, p≤0.01, η2=0.83 and range of motion F (2.29, 29.81) = 51.81, p≤0.01, η2=0.80 main effects were observed. Post hoc analyses revealed that sagittal plane peak angles and ROM using the YXZ and ZXY sequences were significantly greater than the others ( Figure 1 , Table 1 ).

In the coronal plane significant peak angle F (1.32, 17.31) = 31.16, p≤0.01, η2=0.71 and range of motion F (1.38, 17.98) = 62.62, p≤0.01, η2=0.83 main effects were also observed. Post hoc analyses revealed that coronal plane peak angles and ROM using the YXZ and ZXY sequences were significantly greater than the others. In addition, it was also observed that peak angles quantified using the XYZ sequence were significantly greater than for the ZYX sequence.

Finally in the transverse plane significant peak angle F (1.23, 15.92) = 61.50, p≤0.01, η2=0.83 and range of motion F (1.65, 21.42) = 50.86, p≤0.01, η2=0.80 main effects were observed. Post hoc analyses revealed that transverse plane peak angles and ROM using the YXZ and ZXY sequences were significantly greater than the others. In addition, it was also observed that peak angles quantified using the XYZ sequence were significantly different than for the YZX and ZYX sequences.

Comparisons between hip angles using the seven different methods revealed very strong correlations for the sagittal plane (R2 =0.90) and moderate-strong correlations for the coronal (R2 = 0.72) plane. However, comparisons between the methods in the transverse plane revealed weak correlations between waveforms (R2 = 0.15). When coronal and sagittal plane angles were correlated, very low correlations were observed when using the helical axis (R2 = 0.001) XYZ (R2 = 0.06), XZY (R2 = 0.07), YZX (R2 = 0.08), and ZYX (R2 = 0.012) techniques indicating minimal extra-sagittal crosstalk. However, when the YXZ (R2 = 0.26) and ZXY (R2 = 0.12) sequences were used there was evidence of planar crosstalk. When transverse and sagittal plane angles were correlated, very low correlations were observed when using the helical (R2 = 0.06) XYZ (R2 = 0.06), XZY (R2 = 0.07), YZX (R2 = 0.08), and ZYX (R2 = 0.011) techniques indicating little crosstalk. However, when the YXZ (R2 = 0.36) and ZXY (R2 = 0.57) sequences were used there was clear evidence of planar crosstalk.

### Knee

**Figure 2:**
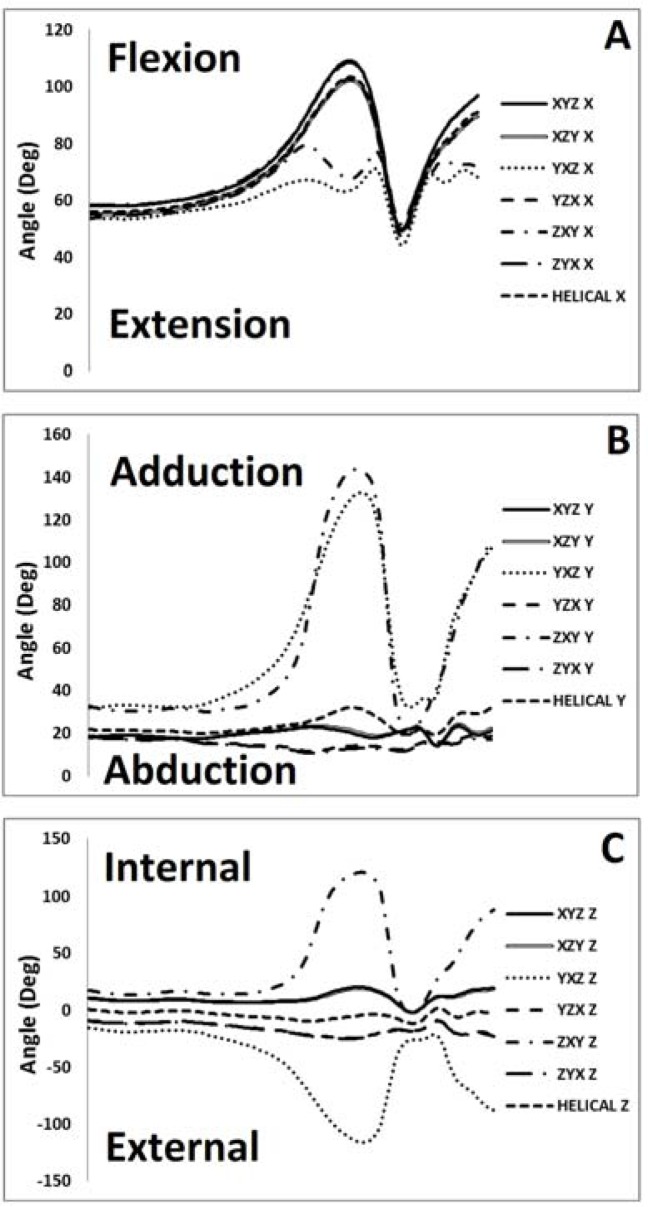
*
Knee joint kinematics in the a. sagittal, b. coronal, and c. transverse plane as a function of Cardan sequence
*

In the sagittal plane a significant main effect F _(1.16, 15.04)_ = 34.99, p≤0.01, η ^2^ =0.73 was observed for the magnitude of range of motion. Post hoc analyses revealed that sagittal plane ROM using the YXZ and ZXY sequences were significantly greater than the others ( Figure 2 , Table 3 ).

In the coronal plane significant peak angle F _(1.60, 20.77)_ = 29.23, p≤0.01, η ^2^ =0.69 and range of motion F _(1.23, 16.00)_ = 48.80, p≤0.01, η ^2^ =0.79 main effects were observed. Post hoc analyses revealed that coronal plane peak angles and ROM using the YXZ and ZXY sequences were significantly greater than the others.

In the transverse plane significant peak angle F _(1.56, 20.16)_ = 9.58, p≤0.01, η ^2^ =0.43 and range of motion F _(1.38, 17.99)_ = 41.44, p≤0.01, η ^2^ =0.76 main effects were also observed. Post hoc analyses revealed that transverse plane peak angles and ROM using the YXZ and ZXY sequences were significantly greater than the others.

Comparisons between hip angles using the seven different methods revealed very strong correlations for the sagittal plane (R ^2^ =0.96 ) and moderate-strong correlations for the coronal (R ^2^ = 0.72 ) plane. However, comparisons between the methods in the transverse plane revealed weak correlations between waveforms (R ^2^ = 0.43 ). When coronal and sagittal plane angles were correlated, very low correlations were observed when using the helical axis (R ^2^ = 0.09 ), XYZ (R2 = 0.01), XZY (R2 = 0.02), YZX (R ^2^ = 0.02 ), and ZYX (R2 = 0.03) techniques indicating minimal extra-sagittal crosstalk. However, when the YXZ (R ^2^ = 0.43 ) and ZXY (R ^2^ = 0.45 ) sequences were used there was evidence of planar crosstalk. When transverse and sagittal plane angles were correlated, very low correlations were observed when using the helical (R ^2^ = 0.01 ) XYZ (R ^2^ = 0.06), XZY (R ^2^ = 0.06), YZX (R ^2^ = 0.08 ), and ZYX (R ^2^ = 0.011) techniques indicating little crosstalk. However, when the YXZ (R ^2^ = 0.36 ) and ZXY (R ^2^ = 0.37) sequences were used there was clear evidence of planar crosstalk.

### Ankle

**Figure 3:**
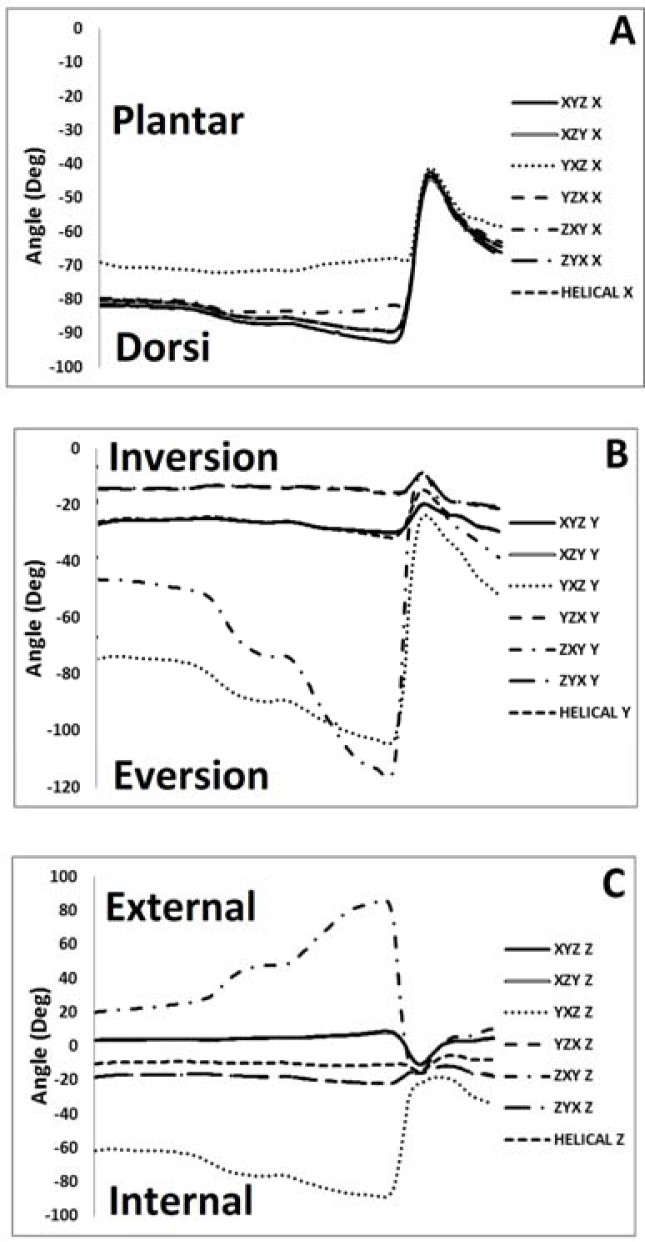
*
Ankle joint kinematics in the a. sagittal, b. coronal, and c. transverse plane as a function of Cardan sequence
*

No significant main effects were observed for the ankle joint in any of the planes of rotation ( Figure 3 , Table 3 ).

Comparisons between ankle angles using the seven different methods revealed very strong correlations for the sagittal plane (R ^2^ =0.97 ) and weak correlations for the coronal (R ^2^ = 0.30 ) and transverse (R ^2^ = 0.35 ) planes. When coronal and sagittal plane angles were correlated, very low correlations were observed when using the helical axis (R ^2^ = 0.009 ), XYZ (R ^2^ = 0.02 ), XZY (R ^2^ = 0.06 ), YZX (R ^2^ = 0.03 ), and ZYX (R ^2^ = 0.04 ) techniques indicating minimal extra-sagittal crosstalk. However, when the YXZ (R ^2^ = 0.70 ) and ZXY (R ^2^ = 0.50 ) sequences were used there was evidence of planar crosstalk. When transverse and sagittal plane waveforms were correlated, very low correlations were observed when using the helical (R ^2^ = 0.06 ) XYZ (R ^2^ = 0.02 ), XZY (R ^2^ = 0.02 ), YZX (R ^2^ = 0.03 ), and ZYX (R ^2^ = 0.05 ) techniques indicating little crosstalk. However, when the YXZ (R ^2^ = 0.71 ) and ZXY (R ^2^ = 0.40 ) sequences were used there was clear evidence of planar crosstalk.

## Discussion

The aim of the current investigation was to determine the efficacy of the different methods of calculating lower extremity 3-D kinematics during the fencing lunge. The analyses of this study represent the first to examine the effect of altering the sequence of rotations during this movement.

The results show that altering the sequence of rotations has a significant influence on the discrete variables obtained in all planes of rotation. This is perhaps surprising and opposes the observations of Sinclair et al. (2012) who suggested that altering the sequence of rotations is insignificant when quantifying rotations in the sagittal plane. There are several potential mechanisms for this observation. Firstly Sinclair et al. (2012) considered only ankle joint kinematics for which no significant differences were observed in the current investigations thus it is likely Sinclair et al. (2012) were overly broad in their conclusion. Secondly the extent of sagittal plane movement during the lunge is considerably greater than during normal running gait ( Novacheck, 1998 ), thus the potential for alterations in the sagittal plane waveforms is accentuated.

In the coronal and transverse planes significant main effects were observed in terms of peak angles and range of motion principally for the YXZ and ZXY sequences. The results indicate that with respect to the hip and knee joint, these sequences were associated with extremely large values for peak angles (YXZ: coronal plane = −102.85°, transverse plane = −96.63° and ZXY: coronal plane = −110.35°, transverse plane = 78.51°) and range of motion (YXZ: coronal plane = 69.54°, transverse plane = 58.43° and ZXY: coronal plane = 118.86° and transverse plane = 112.05°). Furthermore, when coronal and transverse plane profiles were correlated with the sagittal plane, the strongest correlations were observed for the YXZ and ZXY rotation sequences indicating that they are most susceptible to planar cross-talk. This concurs with the observations of Lees et al. (2010) and Sinclair et al. (2012) , who also observed that these sequences were associated with the greatest degree of error. Such was the extent of the planar cross-talk that the resultant waveforms were anatomically impossible, highlighting the extent of the error associated with these sequences. This leads to the conclusion that these sequences cannot be utilized to accurately interpret hip and knee joint function outside of the sagittal plane during the fencing lunge.

That the YXZ and ZXY were associated with the greatest degree of planar cross-talk is an interesting observation as placing X second in the order of rotation appears to be associated with the greatest degree of error. The results of the current investigation support the existence of planar crosstalk, and thus agree with the conclusions of Kadaba et al. (1990) , Thewlis et al. (2008) , Lees et al. (2010) and Sinclair et al. (2012) . The findings from this study do however appear to oppose those noted by Areblad et al. (1990) , who described that varying the ordered sequence of segmental rotations had minimal influence on the resultant angular parameters.

The helical axis technique appears to be relatively stable in terms of its angular outcomes and its lack of sequence dependency is evidenced by the lower values for planar cross-talk which has led some researchers to propose its utilization as an alternative to the Euler angle method ( Hamill and Selbie, 2004 ). However, the susceptibility of this method to noisy data and its sensitivity to the amount of segmental rotation ( Woltring et al., 1985 ), in conjunction with the inability to define a meaningful segment anatomical co-ordinate axis frame, suggest that the representation of 3-D segmental rotations may be negatively affected. This leads to the conclusion that the limited utilization of this technique within sports biomechanics may in fact be warranted.

It is clear from the results that different computational methods can yield different angular kinematic patterns. Utilization of YXZ and ZXY sequences was associated with the strongest correlations from the sagittal plane thus their utilization is discouraged. Observation of the angular profiles and statistical data suggests that using the XYZ sequence to calculate coronal and transverse plane kinematics appears to cause minimal crosstalk from the sagittal plane. Based on these results, it appears that at the current time the ISB recommendations are appropriate for the representation of lower extremity lead leg kinematics during the fencing lunge, and as such its use is encouraged.

## Figures and Tables

**Table 1:** Hip joint kinematic parameters (means and standard deviations) as a function of Cardan sequence (* = significant main effect) .

	** XYZ **	** XZY **	** YXZ **	** YZX **	** ZXY **	** ZYX **	** Helical **
** Sagittal plane **							
Peak angle	102.36 ± 13.56	94.47 ± 11.28	50.23 ± 11.32	95.73 ± 14.33	80.90 ± 6.27	99.15 ± 10.35	91.80 ± 11.10
ROM	67.18 ± 18.31	49.39 ± 10.74	14.97 ± 10.90	58.40 ± 14.05	32.32 ± 14.58	56.39 ± 12.33	53.94 ± 12.24
** Coronal Plane **							
Peak angle	−47.49 ± 10.34	−48.65 ± 10.24	−102.85 ± 15.69	−25.39 ± 13.83	−110.35 ± 62.44	−20.52 ± 11.28	−43.59 ± 10.63
ROM	14.61 ± 12.05	14.99 ± 9.85	69.54 ± 13.94	10.16 ± 7.60	118.86 ± 48.40	8.48 ± 6.53	18.74 ± 10.76
** Transverse plane **							
Peak angle	13.72 ± 15.43	9.93 ± 11.14	−96.63 ± 17.85	−46.76 ± 10.61	78.51 ± 68.60	−47.91 ± 10.79	−33.91 ± 13.13
ROM	30.34 ± 17.81	24.38 ± 12.15	58.43 ± 16.59	14.89 ± 7.62	112.05 ± 44.43	14.89 ± 7.81	7.45 ± 6.48

**Table 2:** Knee joint kinematic parameters (means and standard deviations) as a function of Cardan sequence (* = significant main effect)

	** XYZ **	** XZY **	** YXZ **	** YZX **	** ZXY **	** ZYX **	** Helical **
** Sagittal plane **							
Peak angle	26.91 ± 12.24	27.86 ± 12.60	26.40 ± 11.45	27.87 ± 11.74	26.63 ± 12.52	26.93 ± 13.00	27.13 ± 12.26
ROM	43.40 ± 14.53	41.55 ± 15.18	23.89 ± 10.44	41.71 ± 14.96	27.99 ± 9.97	43.23 ± 14.79	41.86 ± 14.94
** Coronal Plane **							
Peak angle	2.19 ± 7.21	2.26 ± 7.46	91.21 ± 48.16 -	−4.83 ± 7.62	−60.05 ± 67.80	−4.54 ± 7.09	−0.24 ± 8.33
ROM	6.14 ± 3.78	6.22 ± 3.94	86.13 ± 38.29	3.84 ± 2.48	90.67 ± 48.06	3.67 ± 2.20	8.46 ± 5.39
** Transverse plane **							
Peak angle	−17.27 ± 12.21	−17.05 ± 12.02	12.27 ± 53.09	−22.03 ± 9.15	41.49 ± 55.83	−22.35 ± 9.27	−19.59 ± 11.66
ROM	8.63 ± 3.15	8.38 ± 3.05	81.83 ± 39.49	7.86 ± 6.05	86.61 ± 49.94	8.06 ± 6.29	3.97 ± 2.43

**Table 3:** Ankle joint kinematic parameters (means and standard deviations) as a function of Cardan sequence (* = significant main effect).

	** XYZ **	** XZY **	** YXZ **	** YZX **	** ZXY **	** ZYX **	** Helical **
** Sagittal plane **							

Peak angle	−45.46 ± 11.19	−46.50 ± 11.59	−44.80 ± 10.94	−46.36 ± 11.55	−45.06 ± 10.76 -	45.61 ± 11.23	−45.73 ± 11.26
ROM	8.64 ± 6.02	8.56 ± 5.58	6.99 ± 5.74	8.69 ± 5.68	7.35 ± 5.62	8.54 ± 3.91	8.55 ± 5.80

** Coronal Plane **							

Peak angle	−2.53 ± 6.59	−2.58 ± 6.73	−0.15 ± 19.12	7.79 ± 4.53	−10.17 ± 30.98	−1.64 ± 5.58	2.33 ± 6.33
ROM	3.58 ± 1.70	3.60 ± 1.71	19.94 ± 31.67	4.39 ± 2.34	21.60 ± 24.89	4.27 ± 2.24	4.13 ± 2.67

** Transverse plane **							

Peak angle	−12.08 ± 4.61	−11.93 ± 4.78	−5.98 ± 19.84	−15.06 ± 6.88	2.80 ± 35.17	−5.33 ± 6.56	−15.55 ± 6.02
ROM	4.30 ± 2.91	4.21 ± 2.84	20.00 ± 31.12	3.41 ± 1.47	21.77 ± 25.10	3.43 ± 1.46	4.14 ± 1.84
